# How to Deal With Darkness: Modeling and Visualization of Zero-Inflated Personal Light Exposure Data on a Logarithmic Scale

**DOI:** 10.1177/07487304251336624

**Published:** 2025-06-28

**Authors:** Johannes Zauner, Carolina Guidolin, Manuel Spitschan

**Affiliations:** * TUM School of Medicine and Health, Department Health and Sport Sciences, Chronobiology & Health, Technical University of Munich, Munich, Germany; † Translational Sensory and Circadian Neuroscience, Max Planck Institute for Biological Cybernetics, Tübingen, Germany; ‡ TUM Institute for Advanced Study, Technical University of Munich, Garching, Germany

**Keywords:** light exposure pattern, modeling, darkness, light logging, dosimeter, objective light exposure, zero inflation, data transformations

## Abstract

Measuring and analyzing personal light exposure has become increasingly important in circadian and myopia research. Very small measurement values in light exposure patterns, especially zero, are regularly recorded in field studies. These zero-lux values are problematic for commonly applied logarithmic transformations and should neither be dismissed nor be unduly influential in visualizations and statistical modeling. We compare 4 ways to visualize such data on a linear, logarithmic, hybrid, or symlog scale, and we model the light exposure patterns with a generalized additive model by removing zero-lux values, adding a very small or −1 log_10_ lux value to the dataset, or using the Tweedie error distribution. We show that a *symlog*-transformed visualization, implemented in *LightLogR*, displays relevant features of light exposure across scales, including zero-lux, while reducing the emphasis on the small values (<1 lux). *Symlog* is well-suited to visualize differences in light exposure covering heavy-tailed negative values. We further show that small but not negligible value additions to the light exposure data of −1 log_10_ lux for statistical modeling allow for acceptable models on a logarithmic scale, while very small values distort results. We also demonstrate the utility of the Tweedie distribution, which does not require prior transformations, models data on a logarithmic scale, and includes zero-lux values, capturing personal light exposure patterns satisfactorily. Data from field studies of personal light exposure require appropriate handling of zero-lux values in a logarithmic context. *Symlog* scales for visualizations and an appropriate addition to input values for modeling, or the Tweedie distribution, provide a solid basis. Beyond light exposure, other time-series data relevant to biological rhythms, such as accelerometry for ambulatory sleep scoring in humans or wheel-running in animal models, exhibit zero inflation and can benefit from the methods introduced here.

Personal light exposure, or the pattern of ocular light levels across time under free-living conditions, has become increasingly important in human health research (Spitschan et al., 2022). In the past two decades, highly controlled laboratory research has shown the relevance of melanopsin-mediated pathways to the central pacemaker and various downstream effectors to regulate key physiological parameters important for well-being, alertness, sleep, and long-term mental and physical health (Brown et al., 2022; Foster, 2020). However, the real-world effects indicated by these insights can only be gauged when connecting the patterns of light people are exposed to under naturalistic conditions with relevant health outcomes. A growing literature of studies indicates that higher levels during the day and/or lower light levels at night are beneficial for sleep and mental and metabolic health (Burns et al., 2023; da Costa Lopes et al., 2024; Didikoglu et al., 2023; Hand et al., 2023; Lok et al., 2023; Wallace et al., 2024; Windred et al., 2024), with more research required for different cultural, geographical, and other environmental conditions, as well as for different subgroups within a population (Miller et al., 2024; Spitschan et al., 2024). Environmental light levels are also relevant in myopia development and progression (Ho et al., 2019; Landis et al., 2018; Morgan et al., 2021; Muralidharan et al., 2021). Light exposure data in circadian and myopia research are commonly collected with small wearable devices attached to a person at eye level, to the chest, or, from actimetry research, placed on the wrist (Danilenko et al., 2022; Honekopp and Weigelt, 2023).

Compared to controlled laboratory conditions, the dynamic range of light levels under free-living conditions ranges from 0 lux at night to more than 100,000 (10^5^) lux under bright daylight. This poses a unique measurement challenge for the light sensors in small wearable devices (Danilenko et al., 2022). Particularly, this lower bound can be problematic for measurement and analysis: Light below the detection threshold of most sensors is not only theoretically possible but a common occurrence in many people’s daily exposure patterns, for example, in light-limited sleep environments. These circumstances make 0 lux a common value in datasets of personal light exposure, much more so than in past laboratory studies employing research-grade measurement equipment. The high dynamic range of natural light exposure, in many cases, necessitates a logarithmic transformation, which is also appropriate since it mirrors how the retinal photoreceptors and downstream neural machinery operate (Do and Yau, 2010; Wolfe et al., 2017).

As zero cannot be logarithmically transformed into a real number, “zero” instances require manual handling in both statistical models and visualizations. There are no standards for how these zero-lux values should be treated during data pre-processing. Without attention, these observations are often silently dropped from the analysis, skew results, and make plots ambiguous. To handle this problem, some studies add a fixed value to all data points prior to transformation (da Costa Lopes et al., 2024; Estevan et al., 2022), remove small values outright (Balajadia et al., 2023; Hand et al., 2023; Peeters et al., 2020; Stefani et al., 2024), or substitute them with a fixed value (Blackwell et al., 2022; Lok et al., 2023). Others do not mention any special handling prior to transformation (Didikoglu et al., 2023; Price et al., 2021). Likely, zero-lux observations are either dropped or there is a consistent dark signal. While some studies reason with measurement accuracy, others offer no rationale. As a whole, it remains an open question how these choices affect modeling, and subsequently, the interpretation of quantitative results.

We argue that treating small values outside the measurement range, particularly zero-lux values, should be intentional, deliberate, and transparent. This study takes a systematic approach to compare several strategies to treat zero-lux illuminance values in personal light exposure datasets for statistical modeling and visualizations. We note that the relevance and methods shown here differ when using derived metrics based on illuminance measurements, that is, not using the raw time series for visualization and modeling. While these metrics are highly relevant, analysis of the time series of measurements itself and the derived exposure patterns is becoming increasingly important. Analyzing these patterns can provide key insights into individual environmental and behavioral influencing factors on personal light exposure (Guidolin et al., 2024a). Treating the zero-lux case deliberately in these scenarios is key. Beyond light exposure, other time-series data relevant to biological rhythms, such as accelerometry for ambulatory sleep scoring in humans (Lee and Gill, 2018) or wheel-running in animal models (Verwey et al., 2013), exhibit zero inflation and can benefit from the methods introduced here.

## Method And Materials

The dataset used in this study is taken from Guidolin et al. (2024b), is available from Zenodo (Zauner et al., 2025b, 2025c), and is integrated in the *LightLogR* software package (Zauner et al., 2025a). It contains data for a week of personal light exposure measurement of a single participant and simultaneous environmental light measurements, taken from a university rooftop close to the participant’s usual surroundings at the horizontal plane and without any shading or obstructions. One exemplary day from this dataset is used for analysis. As the measure of personal light exposure, we use the melanopic equivalent of daylight illuminance (mel EDI) (CIE, 2018), which is part of the dataset. R statistical software (R Core Team, 2017) is used for analysis with the software package *LightLogR* (v0.5.3) (Zauner et al., 2025a) for data import and visualization. The visualization bases are taken from the *LightLogR* tutorial “The whole game” (https://tscnlab.github.io/LightLogR/articles/Day.html). The light exposure pattern is modeled with a generalized additive model (Wood, 2017) with the *mgcv* package (v1.9-1) (Wood et al., 2017), including the autoregressive error correction for lag 1 (AR1). The analysis documentation and the analysis source code are available as part of a Quarto HTML document (Zauner et al., 2025c).

### Zero-Inflated Data

We refer to zero-inflated data in the context of personal light exposure data. This term is most often used in the context of excess zero values for count data, where zeros exceed the prediction of a Poisson error model (Tu, 2001). However, zero inflation is also appropriate in other contexts where observations of zero-lux are frequent and have to be treated with special care (Tu, 2001), which is the case in this topic. Other cases of zero-inflation are abundant in biological rhythms, such as in sleep-wake cycle data (no activity in the sleep phase), hormonal pulsatility in endocrine rhythms (no secretion of hormones), locomotor activity (no movement), or feeding behavior (no food intake at a given time). Depending on the measure of interest, the data might be continuous (such as activity through accelorometry), or might be count data (event-based data, e.g., activity counts), thus informing the appropriate statistical model.

### Tweedie Distribution

**Figure 1.**
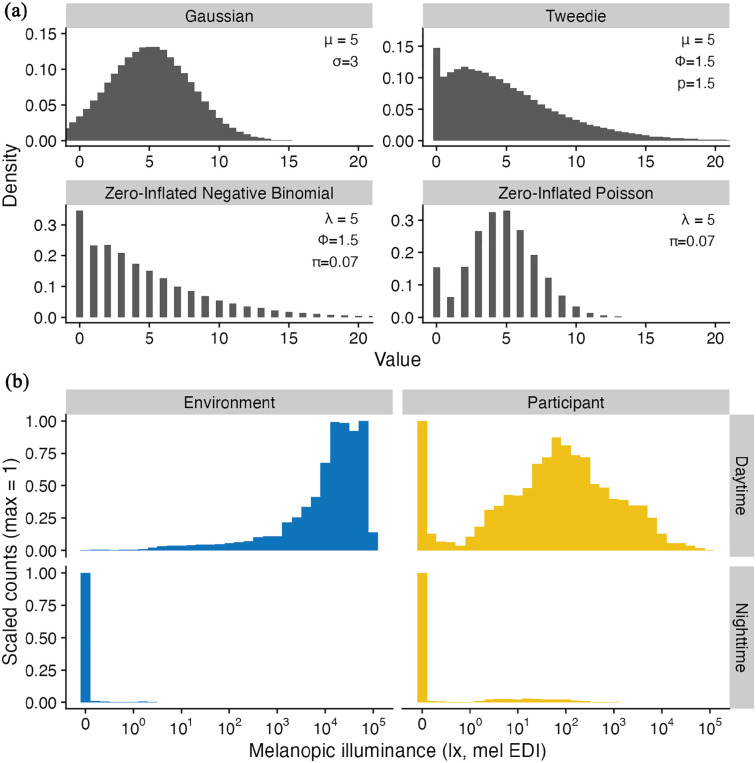
Comparison of statistical distributions and light exposure data. (a) Example distributions for 4 statistical error families. All plots are based on 10^5^ random samples and a binwidth of 0.5 for plotting, with parameter settings as shown in the respective panel. Tweedie refers to the compound Poisson-Gamma distribution used for values of p between 1 and 2 in the Tweedie family. The parameters and plot settings were specifically chosen to highlight the distributions as values approach and meet zero. Gaps in the distribution for zero-inflated negative binomial and Poisson indicate that these distribution functions can only generate integer values. (b) Histograms of light measurements (5-minute intervals) for 1 continuous week, shown on a symlog scaled x-axis. Daytime is calculated from civil dawn until civil dusk and nighttime vice versa. The left panels show measurements of ambient melanopic EDI in the environment, taken at a rooftop. The right panels show measurements taken at a participant’s eye level. Left and right panels contain data from the same general location (same city) and were taken concurrently. The excess of zeros is clearly visible during nighttimes in both cases. However, zeros are also prominent in daytime light levels for the participant.

The Tweedie distribution (Gilchrist and Drinkwater, 1999) is a family of error functions for statistical models that can be used to model continuous positive data that also include zeros. Compared with a (default) Gaussian error model, Tweedie operates on a logarithmic link function, thus integrating the logarithmic nature of light exposure data, and also taking the mean-variance relationship into account. Compared with a zero-inflated Poisson or negative binomial model, Tweedie allows non-integer values. Besides an estimate for the mean, models using the Tweedie family must also set a power parameter p to decide which specific distribution of the family is used. p determines the mean-variance relationship alongside a dispersion parameter. Importantly, if p is 1, a Poisson distribution is used. When p is 2, it corresponds to the Gamma distribution. Between 1 and 2 is the real application of the Tweedie distribution for our purpose, modeling continuous positive values with a spike at zero. Light exposure data fall into the last category, with the example data we are using in this study exhibiting a best fit for p of 1.73 and slight overdispersion with a dispersion parameter ϕ of 1.5. Figure 1 shows a comparison of the most relevant distributions in our context for exemplary parameters in panel A and light exposure data in panel B.

### *Symlog* Transformation

One of the scale transformations used for visualization is the *symlog* scale, or symmetrical log scale. *Symlog* is a logarithmic transformation allowing positive, negative, and zero input values (equation (1)). Positive values are logarithmically transformed. Negative values are logarithmically transformed through their absolute value, with a negative sign applied afterward. A range between a freely defined positive value and its negative counterpart is excluded from logarithmic transformation. *Symlog* scales are available in Python as part of the *matplotlib* library (Hunter, 2007) and in R as part of the *LightLogR* package (Zauner et al., 2025a).

(1) $$f\left(x\right)=\;\{\begin{array}{c} sign\left(x\right)\cdot log\left(\left|x\right|\right),\\ x, \end{array}\begin{array}{c} \left|x\right|>T\\ \left|x\right|\leq T \end{array}$$

### Smallest Non-Zero Value

One of the treatments for non-zero values is to replace them with the smallest possible value ε so that 1+ε≠1 (equation (2)) is true. ε depends on both hardware and software. In R, this value is stored in the object . *Machine$double.eps* and is $2.20446*10^{-16}$ on the machine where the analysis was performed (MacBook Pro with a M4 Max processor, running macOS 15.1.1).

### Statistical Modeling

Table 1 summarizes the treatments of zero-lux measurements explored for statistical modeling.

**Table 1.** Model specifications.

Model	Description	Modification of Mel EDI	Model Formula
1	Removing zero-lux observations	ifmelEDI=0 $then\;mel\;EDI_{1}=NA$	$log_{10}\left(mel\;EDI_{1}\right)\sim \;type+$ s(time,by=type,bs="cc,"k=24)
2	Adding a very small, non-zero value	$mel\;EDI_{2}=$ melEDI +smallestfloat	$log_{10}\left(mel\;EDI_{2}\right)\sim \;type+$ s(time,by=type,bs="cc,"k=24)
3	Adding −1 log_10_(lux)	$mel\;EDI_{3}=$ melEDI+0.1	$log_{10}\left(mel\;EDI_{3}\right)\sim type+$ s(time,by=type,bs="cc,"k=24)
4	Using the Tweedie error distribution	$mel\;EDI_{4}=mel\;EDI$	$mel\;EDI_{4}\sim \;type+$ s(time,by=type,bs="cc,"k=24)

The modification of mel EDI specifies how input values to the model are transformed prior to modeling. The model formula specifies the input formula for the additive model in Wilkinson notation. type specifies that mel EDI can, on average, be different between the participant and the environment (daylight). s() denotes a specification of a smooth relationship between the dependent variable and the independent variable inside the brackets, time in this case, provided as seconds from midnight. The by=type specification fits a smooth relationship for each participant and environment. A basis spline *bs* = “*cc*” bs="cc" specifies a cyclic cubic regression spline, forcing the spline to connect on both ends in its value and its first and second derivative, thus taking into account the cyclic nature of daily light exposure. k=24 specifies the knots, defining the maximum number of basis splines that add up to the smooth relationship.

We chose these approaches with the following rationale:

- **Model 1:** This approach is most conservative from a measurement perspective, as these zero-lux values are almost certainly outside the measurement range of a wearable device.
- **Model 2:** This approach removes the issue of non-real logarithmically scaled values by adding the smallest possible value ε, thus changing the values minimally (~2 × 10^−16^).
- **Model 3:** As Model 2, but without creating a gap in the value range on the logarithmic scale. 0.1 lux is one order of magnitude (−1 log_10_ lux) below the lower measurement range specified by the manufacturer (1 lux).
- **Model 4:** This approach does not include a treatment of the data but consists of the use of the Tweedie distribution (Gilchrist and Drinkwater, 1999).

## Results

### Visualizations

**Figure 2.**
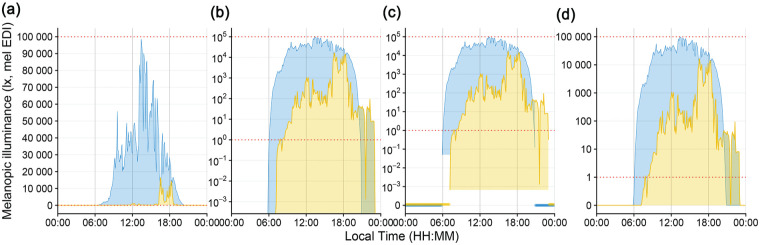
Scaling types to visualize light exposure patterns. All figures show melanopic EDI (lux) on the y-axis and local time on the x-axis. Yellow indicates the personal light exposure of a participant and blue the daylight potential in the area. Red dotted lines indicate the upper and lower measurement range provided by the manufacturer. (a) Linear scaling. (b) Logarithmic scaling with base 10. Zero-lux values are transformed to minus infinity and are not shown. (c) Logarithmic scaling with base 10. Zero-lux values are shown at the bottom of the plot. The lower cutoff for participant and environmental data indicate the minimum measurement value recorded by the device. (d) *Symlog* scaling, with logarithmic scaling, base 10 above 1 lux, and linear scaling below.

Figure 2 shows multiple ways to represent zero-lux measurements as part of a personal light exposure visualization. These are:

A. no scaling
B. logarithmic scaling
C. logarithmic scaling with a separate indicator for zero values (hybrid plot)
D. *symlog* scaling

**Figure 3.**
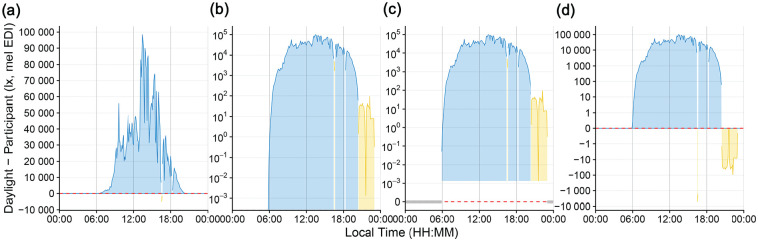
Scaling types to visualize differences in light exposure patterns. All figures show melanopic EDI (lux) on the y-axis and local time on the x-axis. Yellow indicates a participant’s personal light exposure value above the concurrent daylight level and blue indicates higher daylight levels compared with the participant’s exposure. Red dashed lines indicate zero difference. (a) Linear scaling. (b) Logarithmic scaling with base 10. Zero-lux differences are transformed to minus infinity and are not shown. (c) Logarithmic scaling with base 10. Zero-lux differences are shown at the bottom of the plot. The lower cutoff for participant and environmental data indicate the minimum difference between the 2 patterns above zero. (d) *Symlog* scaling, with logarithmic scaling, base 10 above 1 lux, no rescaling between −1 and 1, and base 10 below 1 (calculated from the absolute difference, followed by a sign flip).

Figure 3 shows multiple ways to visualize differences between different sources of light exposure. In this case, this refers to the difference between environmental daylight conditions and a participant’s personal light exposure; however, the principles are valid for differences of light exposure in general.

### Statistical Modeling

**Figure 4.**
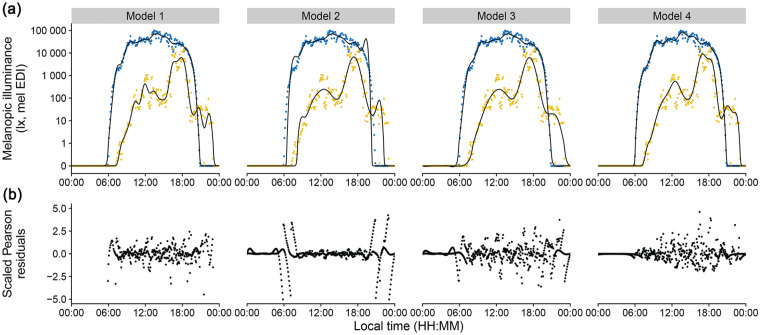
Model results and residual error distribution. a, Personal light exposure on a *symlog* scale for participant (yellow) and environmental light levels (blue). Points indicate measurements; black lines indicate the fitted model. (b) Scaled Pearson residuals/errors for all models.

**Figure 5.**
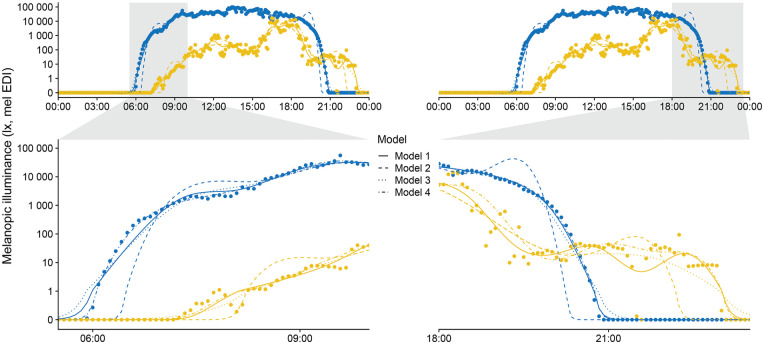
Combined Model results. Personal light exposure on a *symlog* scale for participant (yellow) and environmental light levels (blue). Points indicate measurements; lines indicate the fitted models. Zoomed facets show timeframes that cover transitions from and to zero-lux light levels.

Figures 4 and 5 show model results with the original data.

## Discussion

### Visualizations of Zero-Inflated Data

Figures 2 and 3 show comparisons of typical (panels a and b) and atypical (panels c and d) ways to visualize light exposure patterns. While panel a shows impressive differences in light level magnitude, it is inappropriate regarding sensation and perception of visual and non-visual systems (Wolfe et al., 2017). Panel b is more appropriate in terms of the physiological receiving system. However, it overemphasizes values below 1 lux, which is neither sensible in the measurement accuracy of wearable devices nor in their relevance for the daily light exposure patterns. In other words, we would argue that a difference of 10^−2^ to 10^−3^ lux is not as relevant as a difference of 10^2^ to 10^3^ lux in daily light exposure patterns, even if the light logger were to accurately measure <1 lux values. While the figure could be capped, for example, at the level of measurement accuracy, it would still be ambiguous about whether the nighttime light levels were measured. In addition, only absolute differences can be plotted (Figure 3b). Panel c removes this ambiguity but is harder to understand intuitively and inherits all other downsides mentioned for panel b. Panel d solves the previously mentioned issues by being clear to read and intuitive to understand, including zero-lux values, and without overemphasizing small values. Furthermore, it maps very well to showing heavy-tailed differences in both sign directions (Figure 3d). Beyond that, every point on the *symlog* scale can be mapped to a value by the viewer, as long as the threshold for logarithmic/linear transformation is provided, which is 1 lux in this case, but can be any value. This differentiates the *symlog* scale from, for example, the *pseudo-log* scale available in R (Hadley et al., 2024). *Pseudo-log* smoothes transitions between logarithmic and linear scale. While this approach allows for the same advantages that *symlog* provides, the smooth transition makes it harder to judge which scaling applies. We thus recommend the *symlog* scale as the preferred scaling for personal light exposure visualizations or similarly distributed measures.

### Statistical Modeling of Zero-Inflated Data

We modeled measures of personal light exposure on a logarithmic scale in four different ways. Specifically, we compared the model fits with the original training data to examine how dealing with zero-lux values influences model performance. While (generalized) additive models (GAMs) are not (yet) in common use for personal light exposure measurements, they are very well-fitted to deal with non-linear relationships typical in many natural and biological systems (Hastie and Tibshirani, 1995; Pedersen et al., 2019; Zauner et al., 2022). GAMs model the smooth relationship of the dependent variable to one or multiple linear or categorical predictors. Here, these are mel EDI as the dependent variable, and the time of day and the type of light exposure (environment or participant) as predictors. Compared to, for example, a generalized linear model, GAMs not only allow comparison of model performance as a whole, but also reveal where the model underperforms along the time series. These issues can be mapped to linear models, provided that they contain a similar composition of value ranges as the problematic areas in the additive model. In a standard analysis, data from more than 1 day or person would be modeled with a GAM to extract common trends or decompose the trend into contributing factors, such as chronotype or age. We limited our analysis to a single day to make the deviation of different models from the data directly visible.

Figure 4 reveals these problematic areas, especially pronounced for Model 2 (adding a very small value to the dataset to remove zero-lux values). These areas are transitional times, where the model must deal with zero-lux values and values of several orders of magnitude above in close proximity. Transitional times are shown in a larger zoom in Figure 5 to provide direct model comparisons. In the following paragraph, we first discuss each model individually and then draw comparisons between all models.

**Model 1** (removing zero-lux values, *R*^2^_adj_ = .949) captures the relevant daylight and participant features. The scaled residuals (Figure 4b) show very few patterns within their overall random dispersion. Model diagnostics, in general, are favorable (see analysis documentation S1 section 4.1). The model performs quite well at zero-lux levels, considering it has no training data available in those instances. Transitional areas show only small deviations in the morning for environmental daylight levels. The greatest weakness of this model is that it is used to fit data outside of the (time) range of available data, which is generally not recommended (Wood, 2017). In addition, it is forced to disregard a substantial portion of actual and useful data that, from the model’s perspective, is indistinguishable from actual missing data.

**Model 2** (adding a very small non-zero value, *R*^2^_adj_ = .964) shows strong deviations from training data at the transitional times, which are obvious when comparing the model fit with the data (Figure 4a) and also the residuals (Figure 4b). The minuscule values at the original zero-lux level skew the logarithmically transformed value distribution. This leads to an overcompensation of the model fit on the other end of the distribution, manifesting as an overshoot. Other model diagnostics are also unfavorable, with strong patterns in the residuals (see analysis documentation S1 section 4.2).

**Model 3** (adding −1 log_10_ lux, *R*^2^_adj_ = .981) captures the most relevant features of the daylight and participant data, with only slight deviations at transitional times. In the morning, this is visible in the direct comparison (Figure 5a), but residuals also reveal small structured deviations in the evening (Figure 4b). Other model diagnostics are good, with acceptable deviations from expected residual behavior (see analysis documentation S1 section 4.3).

**Model 4** (Tweedie error distribution, *R*^2^_adj_ = .917) balances pattern features in daylight and participant data and shows no deviations in the transitional periods or structured residual patterns. As a generalized model family, Tweedie is unsupported for the AR1 autoregressive error correction for additive models. As such, while other model diagnostics are good, there is a small amount of autocorrelation in the residuals (see analysis documentation S1 section 4.4).

In summary, if zero-level values are removed (Model 1), model fitting is acceptable only in the scenario when few zero-lux instances exist, but not otherwise. Adding a common value across the dataset to make logarithmic values calculable (Model 2 and 3) skews logarithmic distributions and can lead to erroneous model fits. However, keeping a small distance on a logarithmic level dramatically reduces this effect, to the point where it performs better than when removing zero-level values outright (Model 3 vs Model 1). We recommend using a value close to the lower bound of the manufacturer-specified measurement range. In our example, we added a −1 log_10_ lux value (0.1 lux) across the dataset for a lower measurement boundary of 0 log_10_ lux (1 lux). However, this procedure adds another step to data preparation and post-modeling, as model coefficients and/or predictions need to be readjusted back to the original value level. These steps can introduce new errors in analysis and reporting, especially for complex analyses. At a minimum, we recommend that the exact value added and the procedural steps must be unambiguously reported. Finally, a data transformation is not necessarily required, as the Tweedie error family includes zero values and models data with a logarithmic link function (Model 4). While not the best model for *R*^2^_adj_, its performance is judged on the actual measurement data. This compares to Models 1 to 3, where *R*^2^_adj_ is based upon data that have already been transformed—and, in the case of Model 1, also pruned. Furthermore, the adherence of Model 4 to the data during the transitional phases is remarkable (Figure 5). In terms of the features it captures, Model 4 also strikes a good balance between a slightly too *wiggly* (Wood et al., 2017) Model 1 (participant data), and the overall best fit, but low on features Model 3. This is especially visible in the peaks throughout the day, which are slightly more pronounced in Model 4 than in Model 3 (Figure 4a). Thus, the Tweedie distribution is appropriate for modeling complex personal light exposure patterns on a logarithmic scale and including zero-lux values without any prior data transformation.

### Limitations

One avenue unexplored in this study is data aggregation until at least one value exceeds 0 lux. While certainly possible in some datasets, we find the loss of granularity to solve the issue of zero-lux values rather undesirable. The solution also does not generalize well, as different datasets would require a different level of aggregation. Some datasets, like the one presently used, would require the aggregation into 6-h parts, thus eliminating most features.

Furthermore, while our analysis systematically examines the consequences of different approaches, how it maps to other studies and analyses depends heavily on the statistical approach and distribution of data. However, it should hold strong for whole-day or longer periods of continuous data collection and (generalized) linear/additive (mixed) models.

The recommended *symlog* transformation is seldom used to visualize light exposure patterns and using it in scientific plots might seem unconventional to some. However, we believe its use is justified by the intuitive insight it allows, provided the scaling is explicitly stated, alongside the threshold where the transitions from linear to logarithmic scale occur.

Finally, our approach handles small or zero-lux values “at face value,” that is, not considering measurement errors. We believe that while values below the specified dynamic range of the wearable device might not be accurate to the same degree as values within, they contain valuable information about the overall light exposure pattern, and removing them outright would come at a loss for the bigger picture.

## Conclusion

Very small measurement values in light exposure patterns, especially zero, are regularly recorded in field studies with wearable devices. These values should neither be dismissed nor be unduly influential in visualizations and statistical models. Common types of visualization fail in at least one of those regards. We demonstrated that a *symlog*-transformed visualization style displays relevant features of light exposure across all scales, including zero-lux. Compared with pure logarithmic scaling, *symlog* reduces the emphasis on small values (e.g. <1 lux), which are less important in field studies that do not focus specifically on dark environments, like the sleep environment. Furthermore, this type of scaling maps very well to visualize differences in light exposure covering heavy-tailed negative values. The symlog scale is already part of the software package *LightLogR* (Zauner et al., 2025a) and can thus be easily integrated into the analysis workflow.

We further showed that a small but not negligible value addition to the light exposure data for statistical modeling allows for acceptable models on a logarithmic scale. Those values should be close to the lower end of the measurement range on a logarithmic scale, such as 0.1 lux for a lower bound of 1 lux, as in our case. We also demonstrate the utility of the Tweedie distribution when modeling personal light exposure data, which does not require prior transformations, models data on a logarithmic scale, and includes zero-lux values. In conclusion, we provided a comprehensive analysis of the strengths and limitations of various approaches for addressing zero values in light exposure data. Our goal is to encourage researchers to approach zero-inflated datasets thoughtfully, make informed methodological choices, and ensure transparency in reporting their approaches.
